# Hydrophilic and Hydrophobic Mesoporous Silica Derived from Rice Husk Ash as a Potential Drug Carrier

**DOI:** 10.3390/ma11071142

**Published:** 2018-07-05

**Authors:** Supakij Suttiruengwong, Sommai Pivsa-Art, Metta Chareonpanich

**Affiliations:** 1 Department of Materials Science and Engineering, Faculty of Engineering and Industrial Technology, Silpakorn University, Sanamchandra Palace Campus, Nakhon Pathom 73000, Thailand; 2 Department of Material and Metallurgical Engineering, Faculty of Engineering, Rajamangala University of Technology Thanyaburi, Pathumthani 12110, Thailand; sommai.p@en.rmutt.ac.th; 3 Department of Chemical Engineering, Faculty of Engineering, Kasetsart University, Bangkok 10900, Thailand; fengmtc@ku.ac.th

**Keywords:** mesoporous silica, surface area, rice husk ash, hydrolysis-ageing time, hydrophobicity

## Abstract

This work describes the preparation of mesoporous silica by the green reaction of rice husk ash (RHA) with glycerol, followed by the modification and the potential use as a drug carrier. The reaction was carried out at 215 °C for 2 h. The solution was further hydrolyzed with deionized water and aged for various times (24, 48, 120, 360, 528 and 672 h) before calcinations at 500 °C for 24 h. Further treatment of prepared mesoporous silica was performed using trimethylmethoxysilane (TMMS) to obtain hydrophobic Mesoporous silica. For all synthesized silicas, silica contents were as high as 95 wt %, whereas organic residues were less than 3 wt %. RHA-glycerol showed the highest specific surface area with smallest pore diameter (205.70 m^2^/g, 7.46 nm) when aged for 48 h. The optimal hydrolysis-ageing period of 120 h resulted in 500.7 m^2^/g specific surface area, 0.655 cm^3^/g pore volume and 5.23 nm pore diameter. The surface modification of RHA-glycerol occurred through the reaction with TMMS as confirmed by FTIR (Fourier-transform infrared spectroscopy). Ibuprofen was selected as a model drug for the adsorption experiments. The adsorption under supercritical CO_2_ was carried out at isothermal temperature of 40 °C and 100 bar; % ibuprofen loading of TMMS modified mesoporous silica (TMMS-g-MS) was 6 times less than that of mesoporous silica aged for 24 h (MS-24h) due to the hydrophobic nature of modified mesoporous silica, not surface and pore characteristics. The release kinetics of ibuprofen-loaded mesoporous silicas were also investigated in vitro. The release rate of ibuprofen-loaded MS-24h was much faster than that of ibuprofen-loaded TMMS-g-MS, but comparable to the crystalline ibuprofen. The slower release rate was attributed to the diffusion control and the stability of hydrophobic nature of modified silica. This would allow the design of a controlled release drug delivery system.

## 1. Introduction

Mesoporous silica materials have many superior advantages in which they can be employed in a wide range of industries such as catalysts, absorbents, nano-carrier for drug, pharmaceutics, insulation materials, rubbers, electronics, reinforced composite in plastics, cosmetics and biomedical and dental materials. Mesoporous silica nanoparticles (MSNs) have recently been considered as promising materials to substitute the conventional drug carrier materials due to their large surface area, the possibility of tailoring the surface functionalities for smart targeted drug carriers [1,2] and the variation of the encapsulation [3]. These nanosized porous silica carriers provide more sustained and controlled drug release or enhanced oral bioavailability [4]. Factors such as particle size, particle shape, surface roughness or surface functionalization are important key features to be tailored for more precise controlled drug delivery system. The surface of MSNs attached with a glycosaminoglycan mimetic to create novel antiviral agents against herpes simplex type 1 and type 2 viruses has been prepared and it was found that the nanoparticles acted as viral entry inhibitors, which appeared to block viral attachment and penetration into susceptible cells [1]. Anticancer drug resveratrol could be effectively loaded or encapsulated inside the mesopores of MCM-48 nanospheres without affecting its bioavailability [3].

Thailand is a rice growing and rice exporting country; around 30 million tonnes of rice were produced in Thailand during 2015–2016 [5]. Rise husks are often burnt for energy recovery, which consequently produces rice husk ash (RHA). RHA obtained in a general rice mill consists of more than 80% silica and high contents of char residues and small traces. It possesses a low specific surface area and pore sizes. The recovery of purer amorphous and reactive silica from RHA can be obtained through low temperature calcinations and chemical treatments [6], hence obtaining silicas with lesser impurities and specific physicochemical properties, i.e., tunable specific surface area and pore sizes, which can be beneficial and value-added in some niche products. Many methods [7] are readily employed for the preparation of the mesoporous silica from RHA, including pre- and post-chemical treatments [8,9,10], calcination, the use of templating agent [11,12,13], and sol-gel method [13]. Chemicals used in sol-gel, ageing time, drying method and calcination conditions are all important factors affecting the pore structures and specific surface area. In particular, the ageing of gel is one of the key steps to ensure the maturity of the gel network, in which the condensation reaction is completed. 

The environmentally benign depolymerization reaction of silicate network and RHA in the presence of glycerol near 200 °C was reported [14]. Reactive gels retained the mesoporous nature after hydrolysis and calcination [14]. In addition, the reactive gels can be further modified chemically, e.g., hydrophobic gels, or alternatively used to synthesize other porous materials [15] and aerogels. The reaction of RHA with other bifunctional alcohols to produce reactive gels has also been studied [16]. This work aimed to employ the use of green solvent and renewable resources to prepare mesoporous silica. The effect of hydrolysis-ageing periods on the physic-chemical properties was investigated. The surface treatment of silica was also carried out. Ibuprofen was selected as a model drug. Several works [17,18,19,20] have been performed on the mesoporous silica as a drug carrier using Ibuprofen as a model substance. Vitale-Brovarone C. and colleagues also investigated SiO_2_–P_2_O_5_–CaO–MgO–Na_2_O–K_2_O–CaF_2_ glass–ceramic macroporous scaffolds prepared using glass powders and polyethylene (PE) particles [17]. They found that the ibuprofen loading and kinetics of MCM-41 silica micro/nanospheres with Fa-GC scaffold were strongly affected by MCM-41 spheres inside the scaffold [17]. Monosized MCM-41 spheres incorporated inside a bioactive glass–ceramic macroporous scaffold SiO_2_–CaO–K_2_O (SCK) have been studied and used as Ibuprofen adsorption and release by Mortera R. and co-workers [18,20]. They reported that the adsorption of ibuprofen conducted via the solution method was threefold higher than that of the MCM-41-free scaffold due to the presence of the ordered mesoporous silica [20]. In our work, the drug adsorption in supercritical carbon dioxide on hydrophilic and hydrophobic mesoporous silica was performed to determine the adsorption capability. Finally, the release kinetics were measured and evaluated.

## 2. Materials and Methods 

### 2.1. Materials

Rice husks (RH) were supplied by a rice mill (Taweepattana Rice Mill) in Nakhon Pathom province, Thailand. Glycerol 99.9% was purchased from Sigma-Aldrich Laborchemikalian, Seelze, Germany. Ibuprofen ((RS)-2-(4-isobutylphenyl) propionic acid) ≥99% was purchased from Fluka, China. Aerosil 200 fumed silica was kindly supplied by Evonik, Bangkok, Thailand.

### 2.2. Preparation of Mesoporous Silica from Rice Husks

The procedure for preparing mesoporous silicas from rich husks (RH) was presented elsewhere [14,16]. Briefly, RH was calcined at 500 °C for 24 h to obtain RHA starting materials. RHA and glycerol (1:10 *w*/*v*) were mixed at 200 ± 1 °C, 2 h. The excess glycerol was evacuated after the reaction. The excess deionized water was added for hydrolysis and the mixture was aged at room temperature for various times (24, 48, 120, 360, 528 and 672 h; referred as hydrolysis-ageing periods). Aged gels were washed with distilled water several times and dried at 105 °C, 24 h. Dried gels were again calcined at 500 °C for 24 h (a product referred to MS-00h; 00 is the hydrolysis-ageing period). The experiment was repeated for a hydrolysis-ageing time of 24 h using Aerosil A2000 (FS) for comparison.

### 2.3. Surface Modification of Mesoporous Silica

The surface modification of MS-24h was performed in a mixture of water/ethanol (25/75 by volume): 0.3 g of trimethoxymethylsilane (TMMS) and 1 g of MS was introduced into 100 mL of solution mixture at 60 °C, 8 h. The product was filtered and washed several times finally dried at 60 °C, 8 h. The product named TMMS-m-MS was kept in a desiccator for further analysis. 

### 2.4. Drug Adsorption

**Figure 1:**
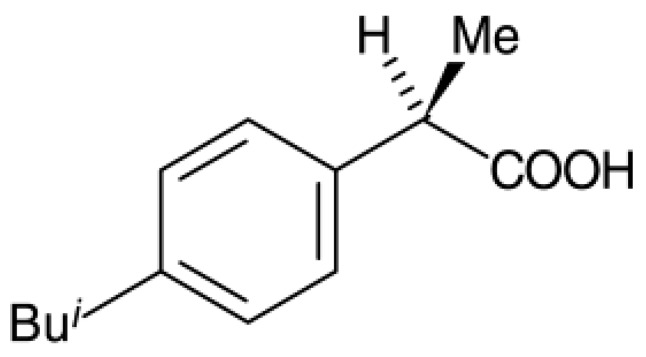
Chemical structure of ibuprofen.

**Figure 2:**
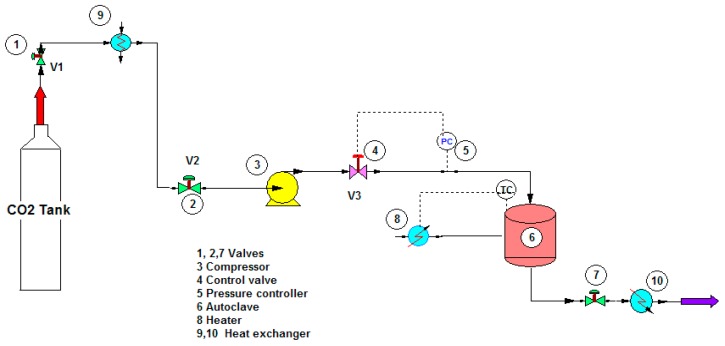
Shemetic representation of the drug loading experiment under supercritical carbon dioxide.

To study the potential use of mesoporous silica, ibuprofen (>99% puris, mw = 206.3 g/mol, Sigma Aldrich) was selected as a model drug. The chemical structure of ibuprofen is presented in Figure 1. Ibuprofen (RS)-2-(4-isobutylphenyl)propionic acid (C_13_H_18_O_2_) is a non-steroidal anti-inflammatory drug (NSAIDs) and has been reported in many studies for drug adsorption under supercritical carbon dioxide due to its good dissolution. For the drug loading or adsorption experiments, 0.05–0.1 g of as-synthesized mesoporous silica (MS and TMMS-m-MS) was weighted into filter paper and placed inside the autoclave chamber as presented in Figure 2. The experimental setup was described by Suttiruengwong S. [21]. Briefly, after closing the autoclave, the preheat carbon dioxide at the temperature of 40 ± 1 °C was fed into the autoclave, where the inside temperature was also 40 ± 1 °C. After that, CO_2_ was slowly fed into the autoclave until the desired pressure (50, 60, 80, 90 and 100 bar) was reached. It should be noted that the critical temperature and pressure of carbon dioxide are 31 °C and 73.7 bar, respectively. The samples were left shaking in the autoclave for 48 h to ensure the equilibrium. CO_2_ gas mixture was then vented out at the constant flowrate. The samples were then weighted again to determine the percent loading or adsorption. The drug loading could be calculated from the increase in weight of the samples. The increase in the weight of the samples indicates the adsorption of drugs in the samples. Alternatively, the concentration of drugs in the samples can be determined by Ultraviolet–Visible (UV-VIS) spectroscopy (λ_max_ = 221 nm, T80+ PG Instruments, Ltd., Leicestershire, UK) using Beer Lambert’s law. The amount of CO_2_ in the autoclave at the equilibrium was calculated from the known volume and CO_2_ density. The values of CO_2_ density were taken from the NIST standard reference database [22]. 

### 2.5. In Vitro Release Experiments

**Figure 3:**
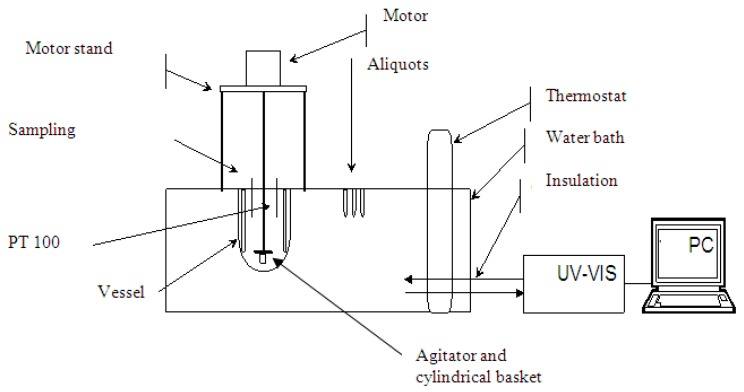
Dissolution experiment assembly.

The accumulative release of ibuprofen was determined using a simulated gastric fluid, 0.1 M HCl (pH 1.2) according to pharmacopoeias (i.e., DAB, USP, Eu. Ph.). The sample (drug crystals or loaded mesoporous silica powder) was weighed and placed in the basket together with filter paper to prevent the loss of the sample powder during the transfer of the basket to the dissolution medium as shown in Figure 3. The amount of the drug was selected so that the sink condition was ensured. The basket was then fixed under the agitator and immersed into a vessel containing 900 mL of dissolution medium (0.1 M HCl) at constant temperature of 37 ± 0.1 °C. The stirring speed was kept at 100 min^−1^. Then, 2 mL aliquots were withdrawn at predetermined time intervals, filtered through a 0.45 m Nylon filter and analyzed using UV-VIS spectrophotometer (Process Instruments Ltd., Burnley, UK).

## 3. Results

### 3.1. Physico-Chemical Properties of As-Synthesized Mesoporous Silica

**Figure 4:**
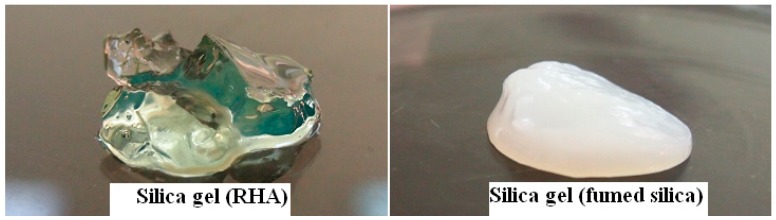
Digital images of gels from RHA (**left**) and fumed silica (FS) (**right**) as starting silica sources.

Fumed silica was used to ensure the reaction of glycerol for comparison. Starting with two different silica sources, fumed silica and RHA, the reaction of glycerol with fumed silica was more favorable than RHA probably due to much higher surface area and high purity of fumed silica. After hydrolysis, the immediately formed gel derived from fumed silica indicated the faster condensation reaction between silanol groups of colloidal silica particles whereas the gel derived from RHA was set after few hours. Figure 4 shows the transparent silica gel prepared from RHA compared to opaque gel prepared from fumed silica. 

Table 1 displays the silica compositions and textural characteristics of starting silica materials (RHA), synthesized mesoporous silica (MS) with various hydrolysis-ageing times, commercial fumed silica (FS) and mesoporous silica obtained from FS (FS-1). The pore characteristics however changed as the ageing time increased to about 48 h. The longer ageing time did not change specific surface area, pore diameter and volume significantly. The maturity of the gels was reached at 120 h as the condensation became very slow. The Brunauer-Emmett-Teller (BET) surface area of MS also increased substantially compared to RHA, whereas in the case of fumed silica, the BET surface area was almost unaffected by this method. However, pore volumes of MS and FS-1 increased. The pore diameter was reduced for MS, but increased in the case of FS-1. The N_2_ isotherm of MS-24h and FS-1 showed a type IV isotherm with H3 hysteresis, suggesting mesoporous characteristics containing slit-shaped pores.

**Figure 5:**
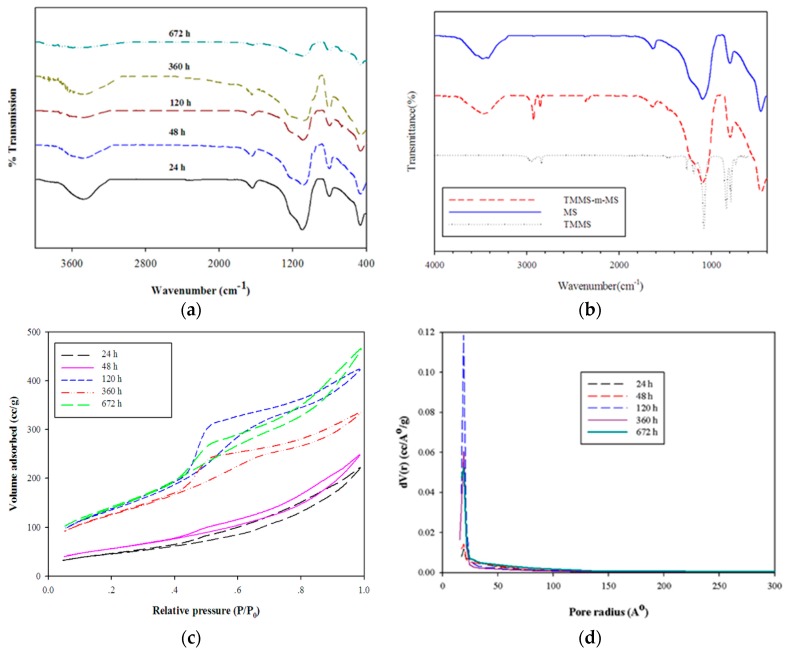
FTIR spectra of mesoporous silica prepared by various hydrolysis-ageing time periods (**a**), surface-modified silica (TMMS-m-MS) (**b**,**c**) N_2_ sorption isotherms of mesoporous silica and (**d**) PSD curves for various hydrolysis-ageing periods.

FTIR spectra (Figure 5a) of the samples hydrolyzed and aged for various time periods indicated the characteristic peak of silica similar to other works [6,21,23]. The characteristic peak of silanol groups and adsorption of water was observed at 3000–3400 cm^−1^. The peak at 950 cm^−1^ was assigned to Si–OH deformation. The asymmetry stretching of Si–O–Si occurred at 1000–1200 cm^−1^. The existence of surface hydroxyl groups could be estimated by normalizing the intensity of Si–OH (I_Si–OH_) at 950 cm^−1^ with the intensity of Si–O–Si (I_Si–O–Si_) at 1100 cm^−1^ as shown in Table 1. A decrease in this ratio indicated the decrease in surface hydroxyl groups. As expected, the samples after calcination at 500 °C for 24 h had hydrophilic characteristics with the presence of silanol groups. The longer hydrolysis-ageing time gave rise to the tendency to reduce silanol groups. FTIR spectra of the surface modification of MS with TMMS as demonstrated in Figure 5b revealed the presence of Si–CH_3_ at 2860 cm^−1^ [24]. The reduction of hydroxyl group intensities around 3300–3500 cm^−1^ also implied that some modification took place. The sample was also observed by floatation on water.

The N_2_ sorption isotherms of mesoporous silicas (Figure 5c) were affected by different hydrolysis-ageing periods. In the hydrolysis-ageing periods were less than 48 h, isotherms consisted of a steep increasing step at the P/P_0_ = 0.1, followed by greater increasing step from P/P_0_ of 0.45, whereas the pore size distribution (PSD) and pore diameter of all samples were insignificantly changed. The shapes of N_2_ sorption isotherms were considered to be type IV with H3 hysteresis loop for samples with less than 120 h of hydrolysis-ageing periods. In hydrolysis-ageing periods >120 h, isotherms exhibited type IV with H3 and H2 hysteresis loops, indicating more complex pores with different sizes and shapes [25]. The gel started to form after hydrolysis, and in the initial hour, the condensation reaction was reversible and exhibited a backward hydrolysis reaction until the optimal time of 120 h was reached. The BET surface area and pore volumes of samples tended to increase whereas the average pore size decreased in the first 48 h. 

**Figure 6:**
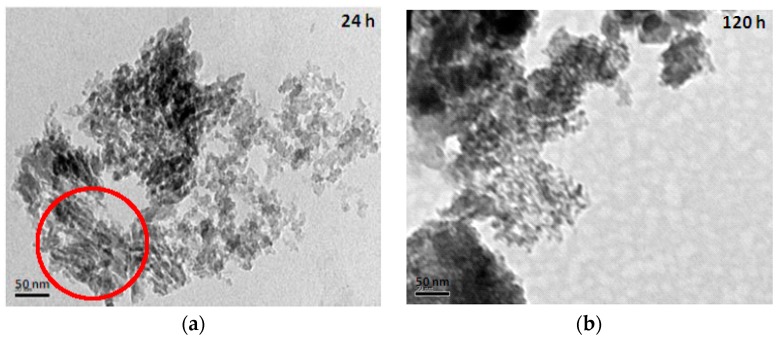

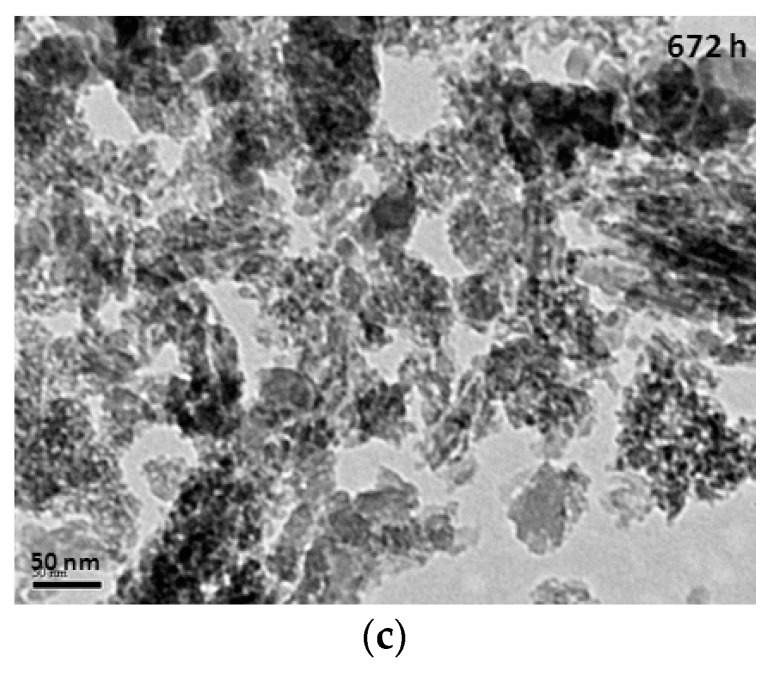
TEM images of mesoporous silica at the hydrolysis-ageing periods of 24 h (**a**), 120 h (**b**) and 672 h (**c**).

The TEM (transmission electron microscope) images of mesoporous silicas with various hydrolysis-ageing periods are shown in Figure 6. Silica particles in the short hydrolysis-ageing period (24 h) exhibited pores with slit shapes or inter-particle void spaces (red circles), where most of the silica particles showed irregularities in shape, whereas, at 120 h of hydrolysis ageing and longer, smaller and denser pores with some existing slit pores were observed. This finding was confirmed with the sorption isotherm analysis.

### 3.2. Drug Loading

**Figure 7:**
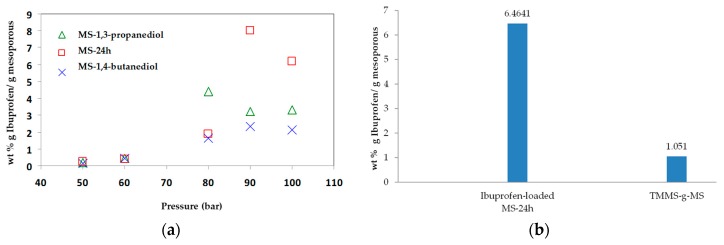
Concentration of (**a**) wt % ibuprofen loaded mesoporous silicas with different pressure at 40 °C and (**b**) ibuprofen-loaded MS-24h and TMMS-g-MS at 90 bar, 40 °C where the equilibrium concentration of Ibuprofen in CO_2_ was 0.0740 wt %.

**Figure 8:**
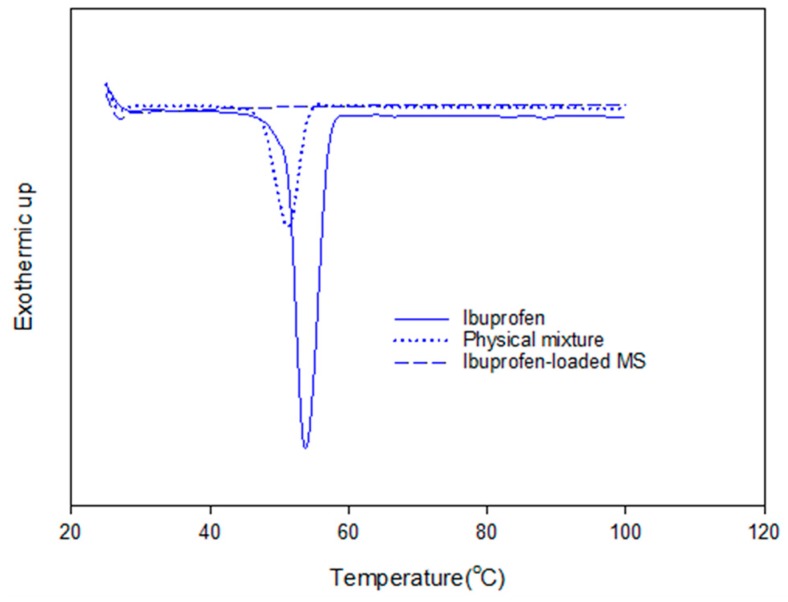
DSC thermograms of crystalline ibuprofen, physical mixture (MS and ibuprofen) and ibuprofen-loaded MS.

The adsorption of ibuprofen under different mesoporous silica derived from glycerol, 1,3-propanediol and 1,4-butanediol was investigated. The synthesized mesoporous silica using 1,3-propanediol and 1–4 butanediol was described elsewhere [16]. The reaction with diols led to more hydrophobic nature as explained by Suttiruengwong S. [16]. From Figure 7a, under supercritical carbon dioxide conditions (above 80 bar), the adsorption of ibuprofen was more than 2-fold higher and was independent of MS type. It should be also noted that these mesoporous silicas had different specific surface areas, pore sizes and pore volumes. MS-24h could take up very high ibuprofen loading, especially at 90 and 100 bar. Therefore, the pressure of 100 bar was chosen for loading MS-24h and TMMS-g-MS. The samples; MS and TMMS-g-MS, were chosen for comparison in order to ensure the effect of the surface modification alone as both samples had fairly similar specific surface area, pore volume and diameter as illustrated in Table 1. Differential scanning calorimetry (DSC) thermograms were recorded for the samples shown in Figure 8. It was observed that the melting peak disappeared for the Ibuprofen-loaded MS sample whereas the typical ibuprofen crystalline and physical mix (ibuprofen and MS mixture) showed melting peaks at 53 °C and 50 °C, respectively. This was evident that the state of ibuprofen after adsorption was amorphous. 

### 3.3. Release Kinetics of Ibuprofen-Loaded Mesoporous Silicas

**Figure 9:**
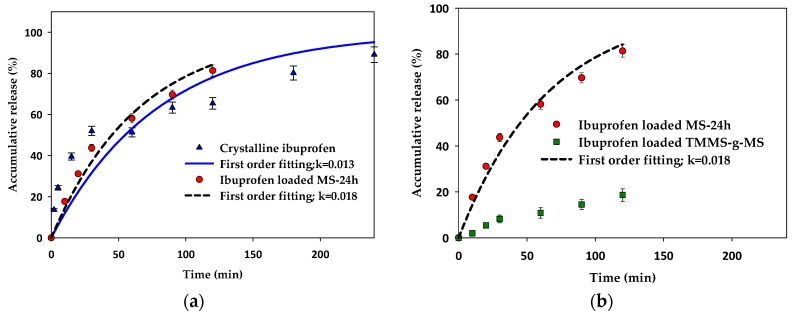
Accumulative release (%) of (**a**) crystalline ibuprofen compared with ibuprofen-loaded MS-24h and (**b**) ibuprofen-loaded MS-24h compared with ibuprofen-loaded TMMS-g-MS with first order kinetics model fitting.

In vitro release tests were carried out for ibuprofen-loaded mesoporous silica and surface modified mesoporous silica aerogels and were compared with the crystalline ibuprofen. The dissolution tests were conducted under the sink condition for all experiments to avoid the solubility effect. As illustrated in Figure 9, the release rate of crystalline ibuprofen was similar to the release of ibuprofen-loaded MS-24h in the first 60 min and became slower after that whereas the release rate of ibuprofen-loaded MS-24h was faster after 60 min. The first order kinetics fit well with the release of ibuprofen-loaded MS-24h. In the case of ibuprofen-loaded TMMS-g-MS (Figure 9b), the release rate was much slower than that of crystalline ibuprofen and ibuprofen-loaded MS-24h. 

## 4. Discussion

The work started with the green synthesis of mesoporous silica using glycerol. The reaction was carried out with two different sources of silica, namely, rice husk ash and fumed silicas. The latter source of silica was very pure, whereas the former one had some trace impurities. The dependency of hydrolysis-ageing time on the physic-chemical properties of as-synthesized mesoporous silicas revealed that the longer the ageing time, the larger the BET surface area. It was found from Table 1 that after reaction and calcination, the silica composition of MS-24h increased from 87 to 95 wt %. All prepared mesoporous silicas were amorphous as analyzed by X-ray diffraction patterns (XRD) (data not shown). Silica contents were increased to 95 wt % and were unaffected by hydrolysis-ageing periods. These organic residues (4–6 wt %) could result from the remaining organic compounds in porous silica structures or incomplete elimination after calcinations [16]. The results showed that the hydrolysis-ageing time influenced the BET surface area, pore volume and pore size. At 120 h of hydrolysis-ageing time, BET surface area reached 500.7 m^2^/g and the pore volume and pore diameter were 0.655 cm^3^/g and 5.23 nm, respectively. FTIR confirmed the reduction of silanol peak intensities, or that more condensation could occur. The surface modification of MS-24h with TMMS showed the hydrophobic nature, but preserved the BET surface area, pore volume and pore diameter.

According to our previous study [21], ibuprofen is highly soluble in supercritical carbon dioxide and high adsorption on porous aerogels was achieved. Such extraordinarily high loadings could indicate that multilayer adsorption or even capillary condensation took place. From Figure 7b, the drug loading of MS-24h was 6 times higher than that of TMMS-g-MS. Ibuprofen has one aromatic ring and a relatively long and flexible hydrophobic moiety (butyl group). As a result, the ibuprofen molecules could favourably pack on the surface, leading to a high adsorption [21]. However, after the surface modification of MS-24h with TMMS, some surface hydroxyl groups were randomly substituted by trimethyl groups. The heterogeneous surface chemistry might be responsible for poor adsorption.

Although the ibuprofen adsorbed on the MS could be in an amorphous state, the release was not significantly enhanced. Smirnova I. and colleagues [26,27] reported that the loading of drugs onto very large surface area of silica aerogels using supercritical carbon dioxide was advantageous in terms of the change of crystalline to amorphous drugs, which was consequently responsible for the faster dissolution and hence release rate. In this case, the effect of dissolution of amorphous ibuprofen was less pronounced. This may be due to the low solubility of ibuprofen in the test media (0.1 M HCl). The release profile of ibuprofen-loaded MS-24h fitted well with the first order release kinetics shown in Equation (1).

ln *C_t_* = ln *C*_0_ + *K*_1_*t* (1)

where *C_t_* is the amount of drug dissolved at interval time *t*, *C*_0_ is the initial amount of the drug in the solution, and *K*_1_ is the first order constant.

In the case of the release kinetics of ibuprofen-loaded TMMS-g-MS, much slower release kinetics of ibuprofen-loaded TMMS-g-MS are observed. This might be due to more hydrophobic nature of this material. Hydrophobic mesoporous silica is more stable in a dissolution medium (e.g., 0.1 M HCl), and pharmaceutical release would be controlled by the molecular diffusion of the drug from the adsorption site (on the surface or in pores) and the penetration of dissolution medium through the porous structure [21]. Thus, the slower release can be expected.

## 5. Conclusions

Mesoporous silica materials at various hydrolysis-ageing periods were prepared from rice husk ash starting materials. High surface area mesoporous silica was obtained at optimal hydrolysis-ageing period of 120 h (500.7 m^2^/g BET surface area, 0.655 cm^3^/g pore volume and 5.23 nm pore diameter). The increase in hydrolysis-ageing periods decreased the size of pores. N_2_ sorption isotherms and TEM images revealed the changes in hysteresis and pore structures. Prepared mesoporous silica was successfully modified by trimethoxymethylsilane. The methyl moiety was responsible for the hydrophobic characteristic. This method provides a sustained route for renewable materials and can potentially be used for various applications. The adsorption of ibuprofen on the mesoporous silcas, MS-24h and TMMS-g-MS, depended on the chemical structure. The % loading of Ibuprofen-loaded MS-24h was 6 times higher than that of Ibuprofen-loaded TMMS-g-MS. The release kinetics of Ibuprofen-loaded TMMS-g-MS was much slower than that of crystalline and Ibuprofen-loaded MS-24h. This was due to the diffusion control of the dissolution medium. The hydrophobic characteristic was more stable in the medium, while in the case of Ibuprofen-loaded MS-24h, the release profile was close to that of crystalline ibuprofen. The slow release rate of ibuprofen-loaded MS will allow for controlled release kinetics.

## Figures and Tables

**Table 1:** Textural characteristics of starting silica, mesoporous silica materials, surface-modified silica materials and fumed silica.

Samples	I_Si–OH_/I_Si–O–Si_ Ratio	Compositions (%)						Surface Area BET (m^2^/g)	Pore Volume (cm^3^/g)	Pore Diameter (nm)
		SiO_2_^a^	K_2_O ^a^	CaO ^a^	MnO ^a^	Fe_2_O_3_^a^	Weight Loss (%) ^b^			
RHA	–	86.74	7.53	2.19	0.23	0.27	3.02	46.26	0.315	27.41
MS-24h	3.628	94.5	0.2	0.3	0.1	0.1	4.84	149.4	0.549	14.69
MS-48h	2.286	95.1	0.2	0.3	0.1	–	4.28	205.7	0.384	7.46
MS-120h	2.070	95.0	0.1	0.2	0.1	–	4.49	500.7	0.655	5.23
MS-360h	1.700	95.5	0.2	0.2	0.1	–	4.06	453.8	0.518	4.56
MS-672h	1.241	95.3	0.2	0.2	0.1	–	4.19	451.9	0.613	6.42
TMMS-m-MS	–	–	–	–	–	–	–	144.3	0.544	14.83
FS ^c^	–	≥99.8 ^c^	–	–	–	–	–	200 ± 25 ^c^	0.338	8.96
FS-1	–	98.9 ^b^	–	–	–	–	1.05 ^b^	208	0.820	24.56

^a^ XRF results after subtraction of weight loss from TGA analysis, ^b^ TGA analysis, ^c^ materials data sheet.
